# Supplementary material for: Laboratory-Controlled Experiments Reveal Microbial Community Shifts during Sediment Resuspension Events
Source: Genes (Basel). 2022 Aug 9;13(8):1416. doi: 10.3390/genes13081416 (PMC9407494; doi:10.3390/genes13081416)
Supplement: Supplementary file 1 [file genes-13-01416-s001.zip › Table S1.pdf]

Table S1. Relative abundance of the major archaeal taxonomic groups, in the oxic (ox) and anoxic (ax) incubations, and in the sediment (S) and water (W) samples. Relative abundance is expressed in percentage of the total number of sequences for each sample (in parentheses). Both numbers indicate abundances for each duplicate. For the water samples, number of sequences per total number of sequences for each sample is given. S0, initial sediment before incubation; Dam, dam lake water sample; Unc., unclassified; Cand., candidatus.

**S0**	Unc. Archaea (21.58-22.05)<br>Bathy_6 (16.84-17.36)<br>Woese_5a (8.1-8.52)<br>Unc. Woesearchaeales (7.65-7.83)<br>*Methanoregula* (5.34-4.92)<br>Woese_5b (5)	**Dam**	Nitrosoarchaeum (6/13)<br>Unc. Archaea (4/13)
**S12H_ox**	*Methanosaeta* (24.47)<br>*Methanosarcina* (23.46)<br>*Methanoregula* (15.68)<br>Rice Cluster 1 (5.53)<br>Bathy_6 (5.02)	**W12H_ox**	Bathy_6 (6/22)<br>*Methanobacterium* (4/22)<br>Cand. Methanoperedens (3/22)<br>*Methanocella* (2/22)<br>unc. Nitrososphaeraceae (2/22)
**S240H_ox**	*Methanosarcina* (23.44-24.67)<br>*Methanoregula* (16.72-12.91)<br>*Methanosaeta* (15.36-15.21)<br>Bathy_6 (9.33-13.86)<br>*Methanocella* (6.31-5.45)<br>Rice Cluster 1 (6.12-5.4)<br>Unc. Methanomassilliococcaceae (5.71-4.73)	**W240_ox**	Bathy_6 (12/71)<br>*Methanosarcina* (10/71)<br>*Methanobacterium* (9/71)<br>Cand. Methanoperendens (6/71)<br>*Methanosaeta* (6/71)<br>Rice Cluster 1 (6/71)
**S720H_ox**	*Methanosarcina* (26.98-24.06)<br>Bathy_6 (17.9-12.86)<br>Rice Cluster 1 (15.36-21.91)<br>*Methanocella* (12.23-15.9)<br>*Methanobacterium* (7.17-6.6)<br>Unc. Methanocellaceae (5.15-2.63)	**W720H_ox**	Bathy_6 (21/113)<br>*Methanobacterium* (18/113)<br>Unc. Nitrososphaeraceae (15/113)<br>Cand. Methanoperendens (12/113)<br>*Methanosarcina* (11/113)<br>*Methanocella* (9/113)<br>Rice Cluster 1 (9/113)<br>*Methanosaeta* (7/113)
**S12H_ax**	*Methanosarcina* (37.34)<br>*Methanosaeta* (14.52)<br>*Methanoregula* (10.44)<br>Rice Cluster 1 (9.28)<br>*Methanobacterium* (6.12)	**W12H_ax**	Cand. Nitrosotalea (1/3)<br>*Methanosarcina* (1/3)<br>Unc. Nitrososphaeraceae (1/3)
**S240H_ax**	*Methanocella* (26.94-24.97)<br>Rice Cluster 1 (21.94-25.08)<br>*Methanosarcina* (19.67-16.1)<br>*Methanobacterium* (15.15-8.48)<br>Bathy_6 (8.31-16.12)	**W240H_ax**	Bathy_6 (7/45)<br>*Methanosarcina* (7/45)<br>*Methanobacterium* (6/45)<br>Unc. Nitrososphaeraceae (6/45)<br>*Methanocella* (4/45)<br>Rice Cluster 1 (4/45)<br>Cand. Methanoperendens (3/45)<br>*Methanosaeta* (3/45)
**W720H_ax**	*Methanosarcina* (64.69-73.35)<br>*Methanocella* (9.31-7.3)<br>Rice Cluster 1 (6.73-7.31)<br>Unc. Methanocellaceae (6.7-3.23)<br>*Methanobacterium* (5.87-3.95)	**W720H_ax**	Unc. Archaea (2/2)
