# Supplementary material for: Laboratory-Controlled Experiments Reveal Microbial Community Shifts during Sediment Resuspension Events
Source: Genes (Basel). 2022 Aug 9;13(8):1416. doi: 10.3390/genes13081416 (PMC9407494; doi:10.3390/genes13081416)
Supplement: Supplementary file 1 [file genes-13-01416-s001.zip › Table S2.pdf]

**Table S2**. Relative abundance of the major bacterial taxonomic groups, in the oxic (ox) and anoxic (ax) incubations, and in the sediment (S) and water (W) samples. Relative abundance is expressed in percentage of the total number of sequences for each sample (in parentheses). Both numbers indicate abundances for each duplicate. S0, initial sediment before incubation; Dam, dam lake water sample; Unc., unclassified; Cand., candidatus.

| | | | |
|---|---|---|---|
| **S0** | *Rhodanobacter* (17.88-18.79)<br>Unc. Acidimicrobiia (10.99-10.73)<br>Unc. Micropepsaceae (9.61-8.07) | **Dam** | Cand. Methylopumilus (10.53)<br>CL500-29 Illumatobacteraceae (8.64)<br>Unc. Xanthobacteraceae (6.96)<br>Unc. Gaiellales (6.39)<br>*Reyranella* (5.13) |
| **S12H_ox** | *Bacillus* (40.87)<br>*Pseudoarthrobacter* (22.13)<br>Unc. Bacillales (6.52)<br>*Massilia* (5.87) | **W12H_ox** | *Sphingomonas* (75.67)<br>*Nevskia* (14.28)<br>*Methylocella* (6.33) |
| **S240H_ox** | *Sphingomonas* (14.96-16.82)<br>*Dyella* (9.98-10.41)<br>*Massilia* (9.49-11.03)<br>*Novosphingobium* (7.74-7.69)<br>*Pseudoarthrobacter* (5.8-5.99) | **W240H_ox** | *Pseudoarthrobacter* (47.68)<br>*Sphingomonas* (24.35)<br>Unc. Micropepsaceae (8.67)<br>*Dyella* (8.47) |
| **S720H_ox** | *Sphingomonas* (11.15-11.61)<br>*Flavisolibacter* (7.1-6.49)<br>*Pseudoarthrobacter* (6.91-8.52) | **W720H_ox** | *Rhodanobacter* (26.72)<br>*Pseudoarthrobacter* (16.81)<br>Unc. Rhizobiaceae (16.4)<br>Unc. Microbacteriaceae (13.91)<br>*Dyella* (8.09)<br>Unc. Micropepsaceae (6.77) |
| **S12H_ax** | *Bacillus* (56.9)<br>*Massilia* (13.53) | **W12H_ax** | *Rhodanobacter* (44.65)<br>*Sphingomonas* (19.72)<br>*Pseudoarthrobacter* (12.55)<br>unc. Burkholderiaceae (6.04) |
| **S240H_ax** | *Clostridium*_sensu_stricto_10 (16.84-17.03)<br>*Clostridium*_sensu_stricto_8 (15.9-16.24)<br>*Bacillus* (13.34-14.01)<br>Unc. Clostridiaceae (8.59-8.87)<br>*Lutispora* (7.94-8.25)<br>*Desulfitobacterium* (5.2-5.06)<br>Unc. Caloramatoraceae (5.15-5.72) | **W240H_ax** | *Pseudoarthrobacter* (23.37)<br>*Magnetospirillum* (19.87)<br>*Sphingomonas* (13.51)<br>*Massilia* (8.38) |
| **S720H_ax** | *Bacillus* (39.48-42.36)<br>*Clostridium*_sensu_stricto_10 (8.75-8.84)<br>WCHB1-32 Prolixibacteraceae (7.16-5.55) | **W720H_ax** | *Flavobacterium* (21.58)<br>*Pseudomonas* (19.77)<br>*Acidovorax* (17.1)<br>*Cellulomonas* (14.2)<br>*Caulobacter* (7.15) |
